# Müller Glia Co-Regulate Barrier Permeability with Endothelial Cells in an Vitro Model of Hyperglycemia

**DOI:** 10.3390/ijms252212271

**Published:** 2024-11-15

**Authors:** Juan S. Peña, François Berthiaume, Maribel Vazquez

**Affiliations:** Department of Biomedical Engineering, Rutgers, The State University of New Jersey, 599 Taylor Road, Piscataway, NJ 08854, USA; jsp222@scarletmail.rutgers.edu (J.S.P.); fberthia@soe.rutgers.edu (F.B.)

**Keywords:** advanced glycation end-products, TEER, anti-VEGF, Transwell assays, junction proteins

## Abstract

Diabetic retinopathy is a complex, microvascular disease that impacts millions of working adults each year. High blood glucose levels from Diabetes Mellitus lead to the accumulation of advanced glycation end-products (AGEs), which promote inflammation and the breakdown of the inner blood retinal barrier (iBRB), resulting in vision loss. This study used an in vitro model of hyperglycemia to examine how endothelial cells (ECs) and Müller glia (MG) collectively regulate molecular transport. Changes in cell morphology, the expression of junctional proteins, and the reactive oxygen species (ROS) of ECs and MG were examined when exposed to a hyperglycemic medium containing AGEs. Trans-endothelial resistance (TEER) assays were used to measure the changes in cell barrier resistance in response to hyperglycemic and inflammatory conditions, with and without an anti-VEGF compound. Both of the cell types responded to hyperglycemic conditions with significant changes in the cell area and morphology, the ROS, and the expression of the junctional proteins ZO-1, CX-43, and CD40, as well as the receptor for AGEs. The resistivities of the individual and dual ECs and MG barriers decreased within the hyperglycemia model but were restored to that of basal, normoglycemic levels when treated with anti-VEGF. This study illustrated significant phenotypic responses to an in vitro model of hyperglycemia, as well as significant changes in the expression of the key proteins used for cell–cell communication. The results highlight important, synergistic relationships between the ECs and MG and how they contribute to changes in barrier function in combination with conventional treatments.

## 1. Introduction

Diabetic retinopathy (DR) is a leading cause of blindness among working adults worldwide, projected to affect more than 160 million people by 2045 [1]. Therapies to treat DR in aging adults face significant challenges, as vision is often diminished via concurrent pathologies that include aberrant angiogenesis, wide-spread retinal hemorrhage, and the accumulation of pro-inflammatory compounds, such as advanced glycation end-products (AGEs) [2]. The complex progression of DR alters the structure and operation of the inner blood retinal barrier (iBRB), a selective neurovascular tissue that meets the high metabolic demands of vision by regulating molecular transport across circulating blood and retinal tissue [3]. Limited understanding of age-related, pathological changes to neurovascular barriers is a significant challenge to the development of effective therapies for diabetic retinopathy.

**Figure 1:**
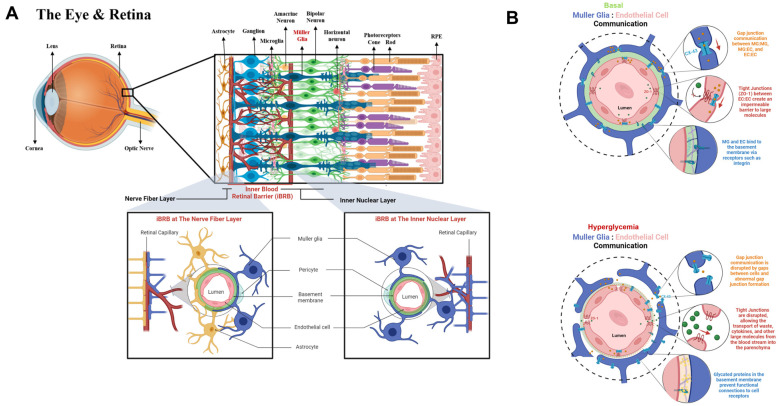
Structure of the inner blood retinal barrier (iBRB) and communication between endothelial cells (ECs) and Müller glia (MG) in basal and hyperglycemic conditions. (**A**) Schematic of the retina and iBRB anatomy. The inner blood retinal barrier mainly comprises endothelial cells, pericytes, a basement membrane, and the foot processes of glial cells. The iBRB is a uniform capillary bed extending from the nerve fiber layer to the inner nuclear layer. The foot processes of astrocytes reside on the nerve fiber layer, while the foot processes of Muller glia are throughout the iBRB. (**B**) MG and EC communication at the iBRB. Muller glia communicate with each other and with endothelial cells via gap junctions formed by connexin 43 (CX-43), enabling the passage of small molecules via juxtracrine communication. Endothelial cells also form tight junctions that selectively regulate transcellular transport across the cell barrier. Particularly, zonula occludens-1 (ZO-1) are the most predominant tight junctions in endothelial cells and their expression is a benchmark for barrier integrity. In diabetic retinopathy (DR), the integrity of the iBRB is compromised and higher permeability across the vascular wall leads to unregulated molecular transport of water, toxins, and cytokines that disrupt cell signaling and lead to neuronal death. Particularly, the expression of gap junctions in MG is disrupted by abnormal junction formation of CX-43. Likewise, the expression of ZO-1 is decreased in diabetic ECs, resulting in higher permeability across the capillary wall.

The iBRB is primarily composed of endothelial cells, pericytes, Müller glia, and astrocytes, as shown in Figure 1A. Endothelial cells (ECs) line the inner surfaces of retinal capillaries and collaborate with pericytes to help regulate angiogenic responses, cell-to-cell communication, and selective molecular transport [4,5]. In complement, astrocyte bodies reside in the retinal nerve fiber layer and extend end feet processes to make direct contact with ECs, while Müller glia (MG) span the entire thickness of the retina and directly communicate with ECs. Tight junction proteins, including occludins, claudins, junctional adhesion molecules (JAMs), and zonula occludens-1 (ZO-1) play a critical role in tight junction formation, which is essential for maintaining the integrity of the inner blood–retinal barrier (iBRB) and its selective permeability to molecules across the vascular wall [6,7]. Heterotypic gap junction proteins, like Connexin-43 (CX-43)—the only gap junction proteins shared between ECs and MG—enable ion balance, waste removal, glucose transport, and direct cell-to-cell communication between these cell types [8], as illustrated in Figure 1B.

In diabetes, chronic hyperglycemia and AGEs accumulation disrupts these critical cell interactions and iBRB responses [9,10]. Notably, AGEs contribute to pericyte death and gliotic scarring, which can require surgical interventions like vitrectomy and astrocyte removal [11]. As a result, MG become key partners for ECs in maintaining iBRB function in aging and diabetic pathology. MG and ECs collaborate through glutamine synthesis in the early stages of DR, where MG convert excess glutamate to glutamine, helping to preserve iBRB integrity by preventing excitotoxicity in ECs [12,13]. Similarly, ECs, in turn, activate protective pathways for MG, promoting cell density maintenance through the VEGFR2-AKT/PKB (Vascular Endothelial Growth Factor Receptor 2–Protein Kinase B) pathway, which upregulates VEGF production under hyperglycemic conditions [14].

As DR progresses, the barrier integrity is further compromised by changes in the tight junction protein distribution. Claudins and JAMs redistribute from cell junctions to the cell surface [15], occludins undergo degradation by matrix metalloproteinases [16], and ZO-1 is downregulated [17]. Similarly, CX-43 expression is downregulated under hyperglycemic conditions but upregulated in response to pro-inflammatory cytokines like IL-1β and TNF-α [18]. Despite these findings, the precise mechanisms through which MG and ECs communicate to regulate molecular transport in hyperglycemia remain poorly understood. Addressing these knowledge gaps is essential for developing targeted therapies that can effectively restore iBRB function and prevent vision loss in DR patients.

This project examined the cellular, molecular, and functional changes in the Müller glia (MG) and the endothelial cell (ECs) barriers in response to the prolonged exposure to high glucose and AGEs typical of hyperglycemia. The conditions stimulated an in vitro, chronic inflammatory state, as evidenced by the increased levels of reactive oxygen species (ROS) and the expression of the receptor for advanced glycation end-products (RAGE), and the cluster of differentiation (CD-40). Dual cell barriers comprising adjacent MG and EC monolayers exhibited greater resistivity—and, thus, greater barrier function—than the cell barriers of the ECs alone. Moreover, the in vitro hyperglycemia model decreased the resistivity of the individual and dual MG and ECs barriers, while treatment with contemporary anti-VEGF compounds restored the resistivity of the dual cell barriers to those of the basal levels. These insights highlight the importance of strategies for treatments that focus on collective barrier responses and their impact with and without conventional treatments, such as anti-VEGF.

## 2. Results

### 2.1. High Glucose and AGEs Induce an In Vitro Hyperglycemic State

Hyperglycemia-conditioned Müller glia (HMG) and hyperglycemia-conditioned endothelial cells (HECs) demonstrated cellular and molecular differences from the MG and ECs when cultured using three different hyperglycemic media conditions of M1: 25 mM glucose and 1 μg/mL AGEs, M2: 25 mM glucose and 5 μg/mL AGEs, and M3: 25 mM glucose and 10 μg/mL AGEs. Tests measured the expression of the reactive oxygen species (ROS), the cluster of differentiation 40 (CD40), and the receptor for advanced glycation end-products (RAGE), i.e., known markers upregulated in diabetic rodent retinal cells in vitro, as previously reported [19,20,21].

### 2.2. Reactive Oxygen Species (ROS) Expression

**Figure 2:**
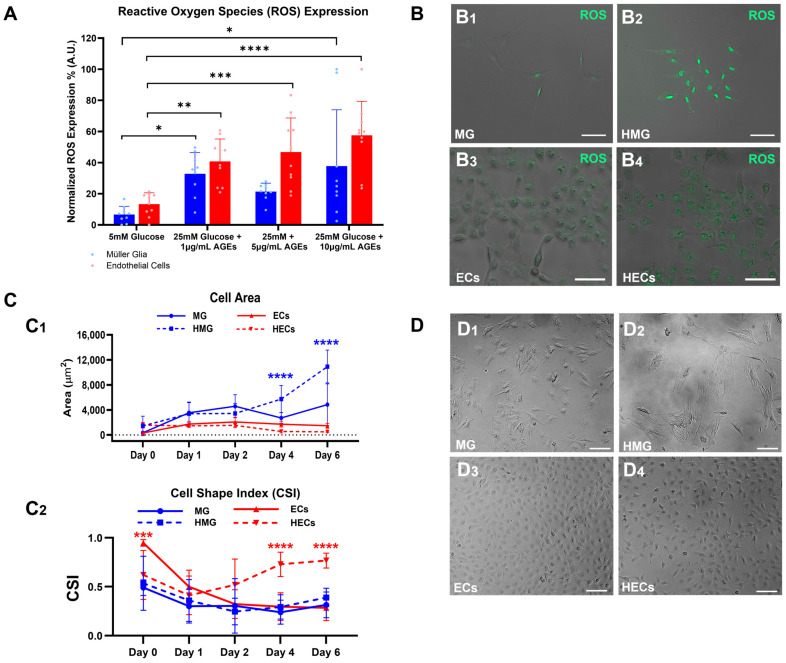
Morphology changes in Müller glia (MG) and endothelial cells (ECs) in response to hyperglycemic conditions formed using high glucose and advanced glycation end-products (AGEs). (**A**) Reactive oxygen species (ROS) expression in MG and ECs in response to different concentrations of hyperglycemic media: 25 mM glucose and either 1 μg/mL, 5 μg/mL, or 10 μg/mL of advanced glycation end-products (AGEs) after 6 days of culture. Ultimately, the condition of 25 mM glucose + 1 μg/mL AGEs was chosen as the hyperglycemic media for this study. (**B**) Representative brightfield and fluorescence images of ROS expression in (**B1**) Müller glia (MG), (**B2**) hyperglycemic MG (HMG)—MG exposed to hyperglycemic media—(**B3**) endothelial cells (ECs), and (**B4**) hyperglycemic ECs (HECs)—ECs exposed to hyperglycemic media—after 6 days in culture with hyperglycemic media. (**C1**) Changes in surface area of MG, ECs, HMG, and HECs in response to control media (5 mM glucose) and hyperglycemic media (25 mM glucose + 1 μg/mL AGEs). (**C2**) Changes in cell morphology over time measured by cell shape index (CSI) of MG, HMG, ECs, and HECs in response to control media (5 mM glucose) and hyperglycemic media (25 mM glucose + 1 μg/mL AGEs. Dots of lighter shade represent individual data points. (**D**) brightfield images of (**D1**) MG, (**D2**) HMG, (**D3**) ECs, and (**D4**) HECs 24 h post-seeding in culture wells. Scale bar is 100 μm. * *p* < 0.05, ** *p* < 0.01, *** *p* < 0.001, **** *p* < 0.0001.

The HMG and HECs demonstrated significantly higher expressions of ROS than the MG and ECs in response to the hyperglycemic media, as shown in Figure 2A. The upregulated ROS expression in the HMG increased 4.9-fold with respect to the MG (6.7% to 32.9%, *p* < 0.05) and in the HECs three-fold (13.4% to 40.8%, *p* < 0.01) with respect to the ECs when exposed to hyperglycemic medium 1, i.e., 25 nM glucose and 1 μg/mL AGEs. Likewise, the ROS expression in hyperglycemic medium 2 (25 nM glucose and 5 μg/mL AGEs) was upregulated in the HMG 3.2-fold (6.7% to 21.4%, n.s.) and in the HECs 3.5-fold (13.4% to 46.8%, *p* < 0.001). Lastly, ROS expression in the hyperglycemic medium 3 (25 nM glucose and 10 μg/mL AGEs) was upregulated in the HMG 5.7-fold (6.7% to 37.8%, *p* < 0.05) and in the HECs 17.1-fold (13.4% to 57.6%, *p* < 0.0001). Despite this, both hyperglycemic medium 1 and medium 3 demonstrated the significant upregulation of ROS for both HMG and HECs; hyperglycemic medium 1 yielded a cell viability above 90%. Hence, hyperglycemic medium 1 was selected for the remainder of this study. Figure 2B shows representative brightfield and fluorescence images of ROS expression in the HMG and HECs on the 6th day post-treatment with hyperglycemic medium 1.

### 2.3. Hypertrophic Changes in Cell Area

The MG cultured in hyperglycemic media 1 (HMG) displayed statistically significant changes in cell area with respect to the MG cultured in basal conditions. These changes illustrate the increased hypertrophy from day 0 (D0 = 1 h post-treatment) to day 6 (D6). The cell area of the MG steadily increased over time in the basal media with a 14.3-fold increase on day 6 with respect to day 0 (D0 = 339.1 μm^2^, D4 = 4837.9 μm^2^, *p* < 0.0001), while the cell area of HMG increased by 7.6-fold (D0 = 1431.8 μm^2^, D6 = 10,937.8 μm^2^, *p* < 0.0001) on the 6th day with respect to day 0. The cell area of the MG and HMG was also significantly different on day 6 (MG = 4837.9 μm^2^, HMG = 10,937.7 μm^2^, *p* < 0.0001), as illustrated in Figure 2(C1). The ECs cultured in hyperglycemic media (HECs) did not display significant changes in cell area with respect to the ECs cultured in basal media over time.

### 2.4. Changes in Cell Shape Index (CSI) of Hyperglycemic Cells

The phenotypic changes in the cell morphology, represented by the CSI, decreased over time for the HMG, MG, and ECs groups. By contrast, the CSI values of the HECs slightly increased, as shown in Figure 2(C2). The average CSI values on day 6 for the HMG (CSI = 0.39 ± 0.09), MG (CSI = 0.31 ± 0.13), ECs (CSI = 0.28 ± 0.13), and HECs (CSI = 0.77 ± 0.08), resulted in significant CSI differences between the HECs and ECs 2.75-fold (*p* < 0.0001). The complete cell area values are shown in Supplementary Figure S1.

The HMG demonstrated remarkable hypertrophic changes in comparison to the MG, as shown in the representative images of Figure 2(D1,D2). The hypertrophy of the HMG was characterized by longer cellular processes with an average of 76.9 μm ± 26.1 μm in length for the HMG and 39.7 μm ± 11.4 μm for the MG. Figure 2(D3,D4) shows the morphology of the ECs and HECs, illustrating smaller cell diameters in the HECs group with an average 20.7 μm ± 3.2, while the ECs displayed an average diameter of 38.1 μm ± 7.4 μm.

### 2.5. Hyperglycemia Induces Changes in RAGE and CD40 Expression

**Figure 3:**
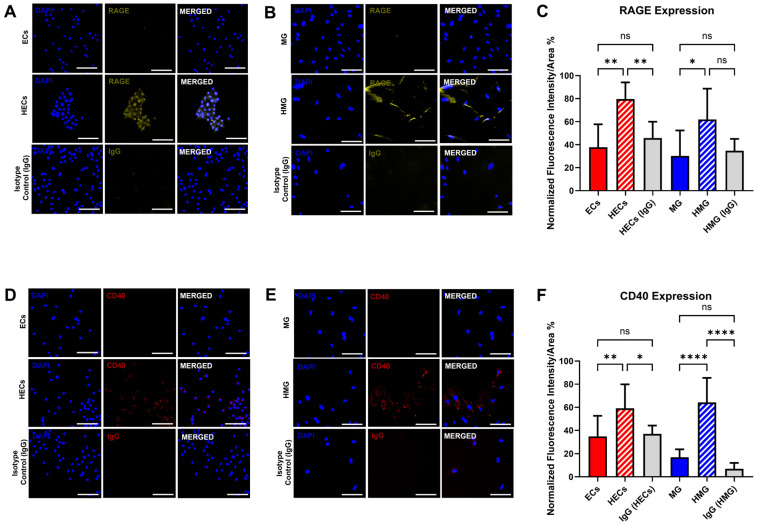
RAGE and CD-40 expression in ECs, HECs, MG, and HMG. RAGE expression in (**A**) endothelial cells (ECs), hyperglycemic endothelial cells (HECs), (**B**) Muller glia (MG), and hyperglycemic-induced Muller glia (HMG) cultured in hyperglycemic media (25 mM glucose and 1 μg/mL AGEs) for 15 days. (**C**) Normalized fluorescence intensity per cell area (%) correlating RAGE expression in all cell groups. CD-40 expression in (**D**) ECs, HECs, (**E**) MG, and HMG cultured in hyperglycemic media (25 mM glucose and 1 μg/mL AGEs) for 15 days. (**F**) Normalized fluorescence intensity per cell area (%) correlating CD-40 expression in all cell groups. IgG was utilized as a negative immunostaining control. Scale bar is 100 μm. * *p* < 0.05, ** *p* < 0.01, **** *p* < 0.0001, ns = not statistically significant.

Tests measured the expression of the RAGE and CD40 in the cell groups via immunocytochemistry. The images of Figure 3A,B show the expression of the RAGE in all the cell groups 24 h post-cell attachment in the well plates. The expression of RAGE in both the HECs and the HMG was significantly higher than the ECs and MG 2.1-fold in both of the groups, as shown in Figure 3C. Note that the fluorescence expression in the isotype control (IgG) showed low background intensity to indicate true RAGE expression in all the other cell groups. The representative images of Figure 3D,E show the expression of CD40 in all the cell groups. The puncta expression of CD40 can be seen as having been significantly upregulated in the HECs and HMG 1.7-fold and 3.8-fold with respect to the ECs and MG, respectively. The fluorescence expression in the isotype control (IgG) for the CD40 expression reveals low background expression, significantly lower than the true expression in the hyperglycemic groups. Figure 3F shows significant differences in CD40 expression in the ECs, HECs, MG, and HMG.

### 2.6. Zonula Occludens-1 (ZO-1) Expression Is Downregulated in HECs

**Figure 4:**
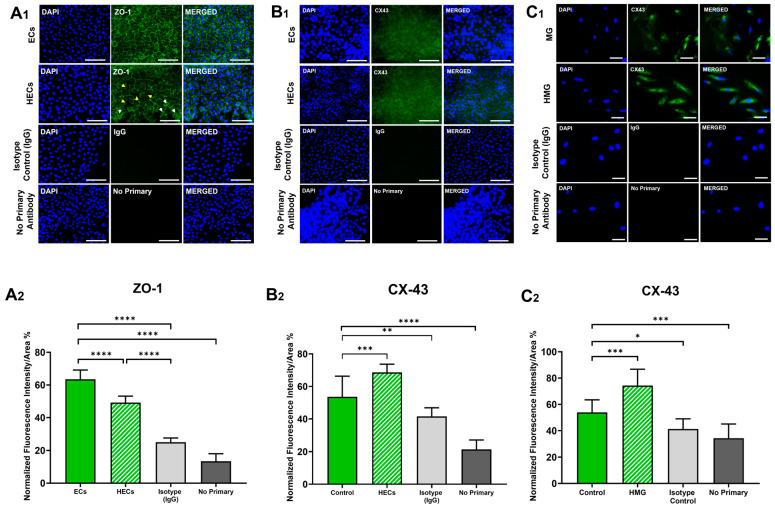
Expression of zonula occludens 1 (ZO-1) and Connexin-43 (CX-43) expression in Müller glia (MG) and endothelial cells (ECs). (**A1**) ZO-1 expression in ECs in response to hyperglycemic condition (25 mM glucose + 1 μg/mL AGEs), and staining controls (isotype control and no primary antibody). Yellow arrowheads point towards the disruption of the ZO-1 boundaries between the adjacent cells. White arrowheads point to clustering of ZO-1. (**A2**) Quantification of ZO-1 in ECs via integrated fluorescence intensity/area. (**B1**) CX-43 expression in ECs in response to hyperglycemic condition (25 mM glucose + 1 μg/mL AGEs), and controls (isotype control and no primary antibody). (**B2**) Quantification of CX-43 in ECs via integrated fluorescence intensity/area. (**C1**) CX-43 expression in MG in response to hyperglycemic condition (25 mM glucose + 1 μg/mL AGEs), and controls (Isotype control and no primary antibody). (**C2**) Quantification of CX-43 in MG via integrated fluorescence intensity/area. Scale bar is 100 μm. * *p* < 0.05, ** *p* < 0.01, *** *p* < 0.001, **** *p* < 0.0001.

HECs demonstrated a 22.4% decrease in ZO-1 expression (*p* < 0.001) when compared to ECs, as seen in Figure 4A. The localization of ZO-1 in HECs the displayed disrupted organization along the cell perimeter, depicting the clustering of ZO-1 in different sections of the monolayer (white arrow heads). ZO-1 was mostly found bordering the perimeters of the ECs. Likewise, gaps in between the HECs were noticeable (yellow arrow heads), caused either by the inability to properly link due to disruption in the process of ZO-1 formation, or by the degradation of the protein causing gaps in the monolayer.

### 2.7. Connexin-43 (CX-43) Is Upregulated in HECs and HMG

The HECs demonstrated a 28.1% higher expression of CX-43 than the ECs (*p* < 0.01), as shown in Figure 4(B1,B2). The expression of CX-43 was observed ubiquitously in the cytoplasm of all the cell groups, as per Figure 4(C1). The expression of CX-43 in the HMG was significantly upregulated by 37.8% (*p* < 0.01) with respect to the control as shown in Figure 4(C2).

### 2.8. Barrier Integrity Varies in Hyperglycemic and Basal Conditions

**Figure 5:**
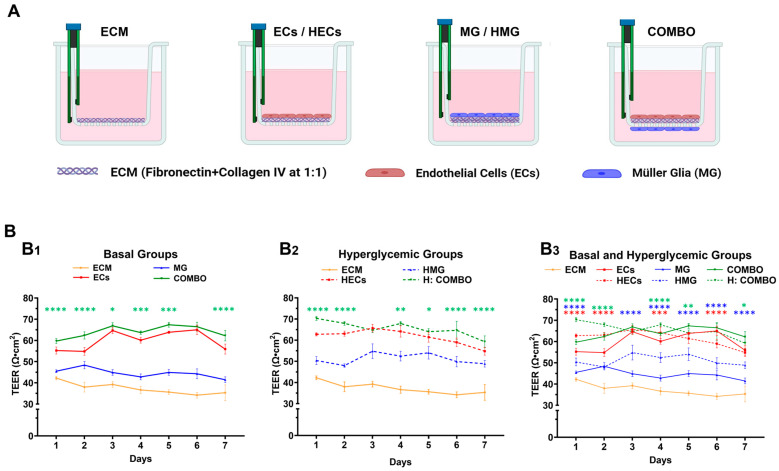
Measurement of barrier integrity via trans-endothelial/epithelial resistance (TEER). (**A**) Schematic depicting the Transwell configurations of cell barriers; ECM: Transwell membrane coated with fibronectin and collagen IV at a 1:1 ratio, 1 mg/mL; ECs: endothelial cells monolayers on coated membranes with ECM. MG: Müller glia monolayers on coated membranes with ECM. COMBOs: ECs monolayer on top of an ECM-coated membrane and MG monolayer on the bottom of the same membrane. (**B**) TEER quantification of (**B1**) normoglycemic and (**B2**) hyperglycemic groups, (**B3**) overlap of normoglycemic and hyperglycemic cell barriers over the course of 7 days. Statistically significant differences between COMBOs and ECs are compared in (**B1**), and between hyperglycemic COMBOs and HECs are compared in (**B2**). Statistical differences between COMBOs and ECs in (**B1**) are represented with green asterisks. Statistical differences between normoglycemic and hyperglycemic groups of the same cell type (e.g., MG and HMG) are compared in (**B3**) using their respective colors for distinction (i.e. blue colored asterisks compare MG and HMG, while red colored asterisks compare ECs and HECs. * (*p* < 0.05), ** (*p* < 0.01), *** (*p* < 0.001), and **** (*p* < 0.0001).

The resistivity of the basal (or normoglycemic) and hyperglycemic cell barriers was assessed using TEER measurements over time. Tests first determined the resistance profiles of the MG, ECs, and COMBOs, as well as the HECs, HMG, and hyperglycemic H-COMBOs by measuring the barrier resistance over the course of 7 days, as per Figure 5A. The differences in the cell barrier resistance among the basal groups are shown in Figure 5(B1), the differences among hyperglycemic the cell groups are shown in Figure 5(B2), and the comparisons between the normoglycemic cell cohorts and the hyperglycemic cohorts are in Figure 5(B3).

As shown in Figure 5(B1), the ECs (red line) displayed a sharp increase in TEER after day 2 and remained steady until a final decrease on day 7. By contrast, the MG (blue line) demonstrated a steady TEER profile over the 7 days with an overall TEER decrease. The TEER values of the ECs monolayer were consistently higher than those of the MG monolayers, and the TEER values of the COMBOs (green line) were higher than both for the full 7 days. Note that the significance is denoted by green stars (*p* < 0.01 to *p* < 0.001) for the COMBO conditions against the ECs, where the TEER values are the closest overall. By contrast, Figure 5(B2) shows a steady decline in the TEER values of the HECs (red dashed line) from day 3 until day 7, while the TEER values of the HMG (blue dashed line) varied by less than 10% over time. Further, the HMG data exhibited an oscillatory pattern of slightly increasing and decreasing TEER values per day. Despite the sharp TEER decline in the HECs, the values remained higher than those of the HMG, with a final 12.1% TEER difference between the two groups on day 7. The hyperglycemic H-COMBOs (dashed green line) also displayed a steady TEER decline from day 4 until day 7, but the values remained above the TEER data of the individual HECs and HMG. On day 7, the TEER values of the H-COMBOs were 8.2% and 21.3% higher than those of the individual HECs and HMG barriers, respectively. Significance is denoted by green stars (*p* < 0.01 to *p* < 0.001) against the H-COMBOs and HECs, as before. In aggregate, Figure 5(B3) illustrates the similar TEER patterns between the HECs and ECs (red dashed versus red solid lines), where both of the groups exhibited increases in barrier resistance until day 3, followed by a steady decreased resistance until day 7. The TEER values of the ECs were slightly higher than the ones of the HECs (~2%). By contrast, the HMG groups displayed an increased barrier resistance during the first 3 days, followed by an oscillating pattern. Surprisingly, the TEER values of the HMG groups were greater by 17.8% than the TEER of the MG groups on day 7 (blue dashed versus blue solid lines). The H-COMBOs exhibited a similar oscillatory TEER pattern as the HMG, with a downward trend after day 4. Note that the green stars denote the significance between the COMBOs and H-COMBO values of the TEER, the red stars denote the significance between the monolayers of the ECs and HECs, and the blue stars denote the significance between the MG and HMG monolayers.

### 2.9. Hyperglycemic Cell Barriers React Differently to TNF-α Treatment than Basal Cell Barriers

**Figure 6:**
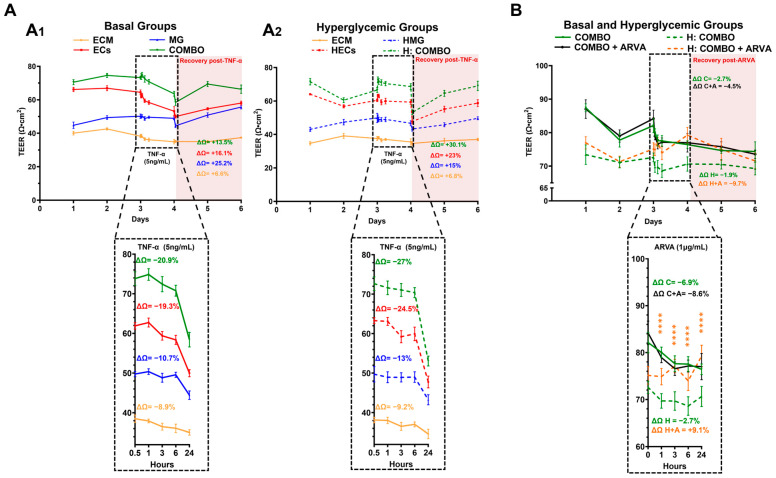
Barrier recovery response to TNF-α and ARVA via trans-endothelial/epithelial resistance (TEER). (**A**) TEER quantification of cell barriers in presence of TNF-α (5 ng/mL) in (**A1**) normoglycemic groups (solid lines) and (**A2**) hyperglycemic groups (dashed lines). ECM: Transwell membrane coated with fibronectin and collagen IV at a 1:1 ratio, 1 mg/mL, ECs: endothelial cells monolayers on coated membranes with ECM. MG: Müller glia monolayers on coated membranes with ECM. COMBO: ECs monolayer on top of CM-coated membrane and MG monolayer on bottom of same membrane. (**B**) TEER quantification of cell barriers in presence of Anti-Rat VEGF-A (ARVA) (1 μg/mL) in normoglycemic groups (solid lines) and hyperglycemic groups (dashed lines). COMBO: ECs monolayer on top of ECM-coated membrane and MG monolayer on bottom of same membrane. Hyperglycemic COMBO: COMBO in hyperglycemic conditions. COMBO + ARVA: COMBOs treated with ARVA (1 μg/mL). Hyperglycemic COMBOs + ARVA: hyperglycemic COMBOs treated with ARVA (1 μg/mL). ΔΩ is percent TEER recovery, measured by TEER change between day 4 and day 6. ΔΩ = COMBOs, ΔΩ C + A = COMBOs +ARVA, ΔΩ D = Hyperglycemic COMBOs, and ΔΩ DC + A = hyperglycemic COMBOs +ARVA. **** (*p* < 0.0001) correspond to the statistical difference between hyperglycemic COMBOs and hyperglycemic COMBOs + ARVA.

The TEER of the EC, MG, and COMBOs confluent cell barriers cultured in basal conditions was measured for 6 days, with and without TNF-α treatment, as shown on Figure 6(A1). During the first 3 days, the COMBO groups displayed the highest TEER values, followed by the monolayers of the ECs, and the MG groups. Upon the addition of TNF-α on day 3, the cell barrier resistance of the ECs exhibited a 19.3% decrease (inset), the MG groups experienced a 10.7% decrease (inset), and the barrier resistance of the COMBO groups decreased by 20.9% (inset) over the course of 24 h. The recovery of the cell barrier resistance after the removal of the TNF-α was measured by calculating the percentage increase in the TEER from day 4 to day 6 in all the cell barrier groups. As shown in the shaded area of Figure 6(A1), the MG cell barriers displayed the highest recovery with a 25.2% increase in the TEER, followed by the ECs with 16.1%, and the COMBO groups with a 13.5% increase.

The TEER data of the hyperglycemic cell barriers is illustrated in Figure 6(A2). The barrier resistance of the hyperglycemic barriers was similar to those of the basal groups with the highest TEER value measured in the H-COMBO groups, followed by the monolayer barriers of the HECs, and the HMG monolayers. As shown in the inset, the addition of TNF-α resulted in a sharp decrease in cell barrier resistance in all of the hyperglycemic groups. The HECs exhibited a 24.5% decrease in the TEER, followed by the HMG with a 13% decrease, and the H-COMBOs with a 27% decrease over the 24 h exposure to TNF-α. In contrast to the basal conditions, the recovery of the hyperglycemic cell barriers post-TNF-α removal was led by the H-COMBO groups with a 30.1% increase in the TEER, followed by a 23% increase in the HEC monolayers, and a 15% increase in the HMG monolayers, as shown in the shaded area of Figure 6(A2).

### 2.10. Anti-VEGF Treatment Increased Resistivity of Hyperglycemic Cell Barriers

The final tests exposed the COMBOs of the cells cultured in the basal conditions and in the hyperglycemic conditions (H-COMBOs) to treatment with anti-VEGF (ARVA) and measured the changes in the barrier resistivity. Note that the groups not denoted with ARVA were treated with a simple change in their respective media. As seen in Figure 6B, the basal COMBO groups (solid line) displayed higher TEER values than the hyperglycemic H-COMBO groups (dashed lines) in the first 3 days, prior to ARVA treatment. However, the treated basal groups (COMBOs + ARVA) displayed a TEER decrease of 8.6% upon exposure to ARVA, while the basal COMBO groups alone exhibited a TEER decrease of 6.9% when treated with a media change (Inset). We note that the inset values highlight the larger change in TEER with respect to each barrier. By contrast, the treated hyperglycemic groups (H-COMBOs + ARVA) experienced a notable and significant 9.1% increase in their TEER, while the H-COMBOs alone exhibited a slight decrease of 2.7% (Inset). Yellow stars denote the significance (*p* < 0.01 to *p* < 0.001) between the TEER values of the treatment groups (COMBOs + ARVA) and (H-COMBOs + ARVA). As shown, the TEER values of the hyperglycemic groups (H-COMBOs + ARVA) after ARVA exceeded those of the basal COMBO group, with and without ARVA. After the media was changed to remove ARVA from all the targeted groups, the TEER values of the (COMBOs + ARVA) group decreased by 4.5% at day 6, while the TEER of the hyperglycemic (H-COMBOs + ARVA) decreased by 9.7%, as shown in the shaded area of Figure 6B. Notably, there were no statistically significant differences in the TEER values between the basal and H-COMBO conditions with ARVA during the recovery period of day 4 through day 6 (ns: *p* > 0.05). Lastly, the TEER of the COMBO and H-COMBO groups untreated with ARVA decreased by a modest 2.7% and 1.9%, respectively, upon media change (ns with *p* > 0.05).

## 3. Discussion

Diabetic retinopathy is a rising health challenge with a lack of therapies to treat its chronic stages in aging adults. Understanding the cellular mechanisms that regulate barrier integrity is critical for the development of treatments to prevent and/or decelerate vision loss. This study highlights that both Müller glia (MG) and endothelial cells (ECs) respond to an in vitro model of hyperglycemia, with significant changes in cell area and morphology. Further, we observed a synergistic relationship between the two cell types in how they contribute to forming barrier function via changes in protein expression and resistivity.

In vitro models of hyperglycemia using high glucose and advanced glycation end-products (AGEs) were able to condition MG and ECs into a pro-inflammatory state. While high glucose has been traditionally used to emulate hyperglycemic environments, recent studies have suggested that AGEs are an important contributor to the long-term effects of hyperglycemia [22]. Our study examined hyperglycemia by using media with high glucose and three different concentrations of AGEs (1 μg/mL, 5 μg/mL, and 10 μg/mL) to illustrate that the AGEs upregulated ROS expression and stimulated significant changes in the cell area and morphology on both the MG and ECs (Figure 2). The selected hyperglycemic condition of 1 μg/mL stimulated comparable ROS upregulation in both the ECs and MG, which is critical for the study of cooperative transport across dual cell barriers. Additionally, the MG exhibited significant hypertrophy associated with glia reactivity [23,24], while the CSI of the HECs showed cytoskeletal rearrangement, an effect observed in the ECs undergoing a pro-inflammatory state [25].

The upregulation of the AGEs receptor, RAGE, in both the hyperglycemia-induced Müller glia (HMG) and the hyperglycemia-induced endothelial cells (HECs) supported a pro-inflammatory state (Figure 3). The RAGE was localized in the HECs’ nuclei, as reported previously in vitro [26,27], but largely seen in the cytoplasm of the HMG, consistent with in vivo studies reporting the RAGE localized in the end feet of MG [21,28]. Moreover, this study is among the first to report the expression of CD-40 in HMG using an in vitro model of hyperglycemia (Figure 3), as observed in diabetic rats [20,29] to highlight the significance of in vitro models using AGEs in addition to high glucose.

Next, our data examined the dysfunction of tight and gap junction proteins between iBRB cells, which are linked to increased vascular permeability [17,30]. ZO-1 was significantly downregulated in the HECs cultured in our hyperglycemia model (Figure 4), consistent with previous in vitro studies [31,32] and with ex vivo studies showing the increased permeability of retinal microcapillaries from diabetic rodents [33]. We additionally examined CX-43 expression, as mounting evidence illustrates its crosstalk with the cytoskeleton, focal adhesion complexes, and other junctional structures helps modulate barrier function (reviewed in [34]). Data illustrated the significant upregulation of CX-43 in HECs and in HMG to highlight the underexplored influences of CX-43 on the communication between and across these cell groups [35,36].

The study next examined the influence of our in vitro hyperglycemia model on the resistivity of individual cell barriers formed of ECs and MG groups, alone, as well as in combination barriers (COMBOs), i.e., dual barriers formed by a monolayer of each cell type. The barrier recorded higher TEER values from the monolayers of the ECs than the MG in both the normoglycemic and hyperglycemic states (Figure 5), consistent with other studies [37,38] that attributed these differences to the downregulation of the tight junctions formed across ECs but absent in MG. However, the barriers of the HMG exhibited surprisingly higher TEER values than the normoglycemic MG barriers, which may be attributed to the intrinsic nature of the MG that serve as the first line of defense against retinal insults [39]. This increase may also be attributed to the MG hypertrophy measured in this study (Figure 2), which increased in surface area and which may potentially promote cell–cell communication. The COMBOs exhibited higher TEER values than either cell monolayer alone, as expected, due to the increased cell density resulting from the bilayer co-culture, a trend consistent with findings in other in vitro neurovascular models [37,40,41]. However, the unexpected observation of the higher TEER in the hyperglycemic COMBOs compared to the normoglycemic COMBOs during the first two days suggests a previously unexplored synergistic interaction between the ECs and the MG. Specifically, the functional pathways associated with the tight junction expression in ECs, including VEGF/VEGFR-2 signaling, Wnt/β-catenin pathways, and Rho GTPase signaling [42], warrant further investigation under hyperglycemic and pro-inflammatory conditions. Our findings are among the first to highlight the underexplored roles of MG in co-regulating barrier resistivity with ECs in hyperglycemic conditions involving AGEs.

The last set of experiments examined the changes in the resistivity of the cell barriers (normoglycemic and hyperglycemic) exposed to inflammatory stimulus via TNF-α and to anti-angiogenic stimulus via ARVA, an anti-VEGF compound. While the TEER values of the dual cell barriers exposed to TNF-α in the COMBOs were higher than those of the H-COMBOs, the MG cell barriers displayed the highest TEER recovery once the TNF-α stimulus was removed (Figure 6A). A surprising 50% increase in the MG resistivity was measured over the barriers of the ECs to suggest a larger contribution of MG to the COMBO resistance than previously explored. As seen, the overall increase in the barrier resistance of the H-COMBOs was higher than the individual monolayers of the HECs or HMG, highlighting a potentially even stronger role of MG in the transporting of molecules across hyperglycemic barriers.

The experiments lastly evaluated the changes in the dual barrier resistance to ARVA, a rat anti-VEGF molecule that functions similarly to bevacizumab [43,44]. These tests are among the first to examine the combinatory influences of anti-angiogenic agents on ECs and MG under both normoglycemic and hyperglycemic conditions. The TEER of the dual barriers cultured in the basal conditions (COMBOs) was initially higher than that of dual barriers cultured in the hyperglycemia model (H-COMBO), consistent with the data. However, upon treatment with ARVA, the H-COMBO groups exhibited robust recovery, with the TEER values comparable to those under the normoglycemic conditions (*p* > 0.05) (Figure 6B). Moreover, the COMBO groups displayed decreases in resistivity when first treated with ARVA (inset), while the H-COMBO groups treated with ARVA showing increased values of TEER to reach the highest levels after ARVA stimulus. This exciting data suggests that anti-VEGF treatments may increase barrier resistance in the conditions of hyperglycemia to increase our understanding of the comprehensive impacts of contemporary pharmacology [45].

However, further long-term studies are necessary to evaluate the sustainability of these effects. In vivo studies and clinical investigations are also needed to validate the therapeutic potential of anti-VEGF drugs in enhancing cell barrier integrity, particularly in chronic hyperglycemic conditions. Ultimately, these findings offer valuable insights into the role of anti-VEGF therapies in restoring vascular integrity, potentially advancing the treatment approaches for hyperglycemia-associated vascular complications.

## 4. Methods and Materials

### 4.1. Müller Glia (MG) and Hyperglycemia-Induced Müller Glia (HMG)

The Müller glia (MG) cells were isolated from the retina of the adult wild-type Sprague Dawley rats using a Papain dissociation kit (Worthington, Lakewood, NJ, USA). In brief, the adult rats were humanely euthanized using CO_2_ asphyxiation following IACUC guidelines. Their retinas were extracted and mechanically dissociated into cell suspension as per protocol [46]. The cells were cultured in Laminin-coated flasks with 88% low glucose Dulbecco’s Modified Eagle Medium (DMEM) with low glucose (5 mM) (Thermo Fisher, 12320, Waltham, MA, USA), 10% Fetal Bovine Serum (FBS) (VWR, 89510-186, Philadelphia, PA, USA), and 2% penicillin/streptomycin (VWR, 97062-806, Philadelphia, PA, USA) for 9 days. The media was changed every day before lineage characterization. The cells were stained for glial fibrillary acid protein (GFAP), glutamine synthetase [9], and cellular retinaldehyde binding protein (CRALBP) to identify the MG via cell-specific markers, as per established protocols [28,46].

Hyperglycemia-induced MG (HMG) are defined as MG cultured in DMEM with high glucose 25 mM (Thermo Fisher, 11965092, Waltham, MA, USA), 1 µg/mL of advanced glycation end-products (AGEs) (Sigma Aldrich, 121800-10MG-M, Milwaukee, WI, USA), 10% FBS, and 2% penicillin/streptomycin for 15 days before the experiments. All the cell cultures were kept at 5% CO_2_ and 37 °C.

### 4.2. Endothelial Cells (ECs) and Hyperglycemia-Induced Endothelial Cells (HECs)

The rat primary retinal microvascular endothelial cells (ECs) (Cell Biologics, RA-6065, Chicago, IL, USA) were cultured in polystyrene flasks with complete endothelial cell medium (Cell Biologics, M1266, Chicago, IL, USA) containing 2% FBS, 0.1% epidermal growth factor (EGF), 0.1% vascular endothelial growth factor (VEGF), and 1% antibiotic/antimitotic solution.

Hyperglycemia-induced endothelial cells (HECs) are defined as primary rat vein endothelial cells (HECs; Cell Biologics, RD-6009, Chicago, IL, USA) cultured in polystyrene flasks with complete endothelial cell medium (Cell Biologics, M1266) containing 1 µg/mL of advanced glycation end-products (AGES) (Sigma Aldrich, 121800-10 MG-M, Milwaukee, WI, USA), 2% FBS, 0.1% EGF, 0.1% VEGF, and 1% antibiotic/antimitotic solution. The cultures were kept at 5% CO_2_ and 37 °C.

### 4.3. Hyperglycemic Media

Based on previous studies [19,33], the HMG and HECs were exposed to three different hyperglycemic media conditions: (M1) 25 mM glucose + 1 μg/mL AGEs, (M2) 25 mM glucose + 5 μg/mL AGEs, and (M3) 25 mM glucose + 10 μg/mL AGEs for 6 days, changing the media every other day. The changes in the cell morphology were measured each day. After 6 days in culture, the expression of the reactive oxygen species (ROS) (Thermo Fisher, C6827, Waltham, MA, USA) and cell viability values from the LIVE/DEAD assays (Thermo Fisher, R37601, Waltham, MA, USA) were used to determine the effect of the hyperglycemic media on the cells. 

### 4.4. Morphology

The cell morphology was assessed via changes in the cell area and cell shape index (CSI), a quantitative metric to measure changes in the cytoskeletal arrangement and cellular polarity [47,48]. The CSI is a dimensionless parameter widely used [49,50] to quantify the roundness of a cell defined in Equation (1). *CSI* is

(1) $$CSI=\frac{4\pi A_{S}}{P^{2}}$$

where $A_{S}$ is the surface area (${\mu m}^{2}$) and *P* (μm) is the perimeter of the cell. The value of the CSI ranges from 0 to 1, where the values close to 1 represent a perfectly rounded cell and the values approaching 0 denote a fully elongated cell.

This study used a liposaccharide (LPS; MilliporeSigma, L2630, Milwaukee, WI, USA) diluted in DMEM at 4 μg/mL as a positive control to induce the cell area changes in the MG and ECs, as per the literature [51,52]. The images of the cells in the wells were recorded at 1 h, 6 h, 12 h, 24 h, 48 h, and 72 h. Cell morphology changes in the hyperglycemia and control groups were performed using the same cell density in the well plates. Note that day zero (D0) values reflect cell morphology 1 h post-seeding.

### 4.5. Junction Protein Expression and Localization

The expression and localization of the ZO-1 and CX-43 was measured using immunocytochemistry. The MG and ECs were seeded in well plates at a concentration of 1.0 × 10^5^ cells/mL in a 24-well plate. After 24 h, the media was changed to DMEM with low glucose and the cells were allowed to grow for 3 days. Media was collected from each culture, filtered (0.2 μm pore), and replaced with DMEM. After 24 h, the media from each culture well was removed and the cells were fixed to measure junction protein expression via immunocytochemistry.

### 4.6. Immunocytochemistry

Briefly, the MG, HMG, ECs, and HECs were seeded in 24-well plates (VWR, 29442-044, Philadelphia, PA, USA) at a concentration of 1.0 × 10^5^ cells/mL and allowed to attach for 24 h. This lower density was chosen intentionally to prevent cellular overcrowding and elevated metabolite concentrations, both of which could independently increase ROS expression. Briefly, the media from each well was removed, and the wells were washed 3 times with Dulbecco’s phosphate-buffered saline (DPBS) (Sigma-Aldrich, D8537, Milwaukee, WI, USA); the cells were then fixed with cold paraformaldehyde at 4% for 5 min. The wells were washed with DPBS for 5 min twice at room temperature. Blocking buffer solution containing 0.05% Triton X-100 (VWR, AAA16046-AE, Philadelphia, PA, USA), 2% donkey serum (VWR, 80054-446, Philadelphia, PA, USA), and 3% BSA (Millipore Sigma, A5611, Milwaukee, WI, USA) in DPBS was added to each well for 15 min at room temperature (25 °C). Following this, the wells were washed twice with DPBS for 2 min, then a primary antibody was added to each well and incubated overnight. The next day, each well was washed 3 times with DPBS for 2 min, followed by the addition of the secondary antibody solution for 1 h at room temperature. The wells were washed with DPBS for 2 min 3 times, before DAPI (1:1000) was added into each well for 5 min at room temperature. Each well was washed with DPBS 3 times for 2 min. The receptor expression was evaluated via fluorescence microscopy (Leica DMi8, NJ, USA). 

The primary antibodies used in this study were as follows: CD-40 receptor (Thermo Fisher, 500-3704, Waltham, MA, USA), CRALBP (Life Technologies, PA5100178, Carlsbad CA, USA), CX-43 (Thermo Fisher, 71-0700, Waltham, MA, USA), GFAP (Life Technologies, PA518598, Carlsbad CA, USA), GS Polyclonal Antibody (Life Technologies, 11037-2-AP, Carlsbad CA, USA), Rabbit IgG Isotype Control (Thermo Fisher, 02-6102, Waltham, MA, USA), RAGE (Thermo Fisher, PA1075, Waltham, MA, USA), and ZO-1 (Abcam, ab216880, Scranton, PA, USA). The secondary antibodies used were Alexa Fluor™ 488 (Thermo Fisher, A-11078, Waltham, MA, USA), Alexa Fluor 568™ (Life Technologies, A-11057, Waltham, MA, USA), and Alexa Fluor™ 488 (Thermo Fisher, R37118, Waltham, MA, USA). 

The fluorescence intensity values for the individual cells were measured using ImageJ (NIH, v1.53k) [53] by calculating the corrected total cell fluorescence (*CTCF*), which adjusts single-cell fluorescence intensity for background fluorescence in the image [54]. The *CTCF* was measured using Equation (2).

(2) $$\mathrm{CTCF}=\left[I_{D}-(A_{C}\times {\bar{F}}_{B})\right]$$

where $I_{D}$ is the integrated fluorescence density (arbitrary units) of a cell, $A_{C}$ is the surface area (μm^2^) of a cell, and ${\bar{F}}_{B}$ is the mean fluorescence background readings (in arbitrary units) surrounding the cell, as outlined in previous studies [55,56,57]. All the groups were normalized to the same cell sample size for these experiments.

### 4.7. In Vitro Testing System

Transwell assays were used to allow the MG and ECs to form individual and dual cell barriers for testing. Transwell inserts with a polyester (PET) membrane (VWR, 29442-082, Philadelphia, PA, USA) of 10 μm thickness and 0.4 μm pore size were coated with a solution of collagen IV (Millipore Sigma, C6745, Milwaukee, WI, USA) and fibronectin (Millipore Sigma, F0895, Milwaukee, WI, USA) to mimic retinal basement membrane. Briefly, collagen IV and fibronectin were diluted in DPBS to a concentration of 10 μg/mL and mixed in a single solution. Approximately 300 μL of this solution was added to the membrane of each insert and left to crosslink over 24 h. Following this, each insert was washed with DPBS and placed in each well with their respective cell media.

The ratio of MG to ECs at the inner blood–retinal barrier has not been determined in mammals. However, studies in mice and non-human primates have shown that Müller glia nearly completely ensheathes retinal capillaries [58]. Based on these findings, we seeded Müller glia and endothelial cells at equal densities and ensured that the Transwell membranes were uniformly covered with confluent cell layers before starting the experiments. Briefly, the MG, HMG, ECs, and HECs were seeded on the top basement membrane-coated membrane individually or in COMBOs (i.e., MG with ECs or HMG with HECs). Single cell barriers were seeded at a concentration of 1.0 × 10^5^ cells/mL and left to form confluent monolayers for 48 h. The COMBO conditions required Transwell inserts to be flipped upside down and a cell solution of either MG or HMG to be added on the bottom side of the insert’s membrane. The cells were left to attach for 1 h, then the Transwell inserts were flipped and placed back in the wells containing the respective media for 24 h at 5% CO_2_ and 37 °C. Following this, the ECs or HECs were added atop the Transwell membranes and allowed to attach and form confluent monolayers for 48 h. Only the confluent monolayers validated via brightfield microscopy were used for the experiments. Additionally, a Transwell insert coated with the basement membrane solution with no cells was used as a control (ECM, only).

### 4.8. Barrier Resistance

The integrity of the cell barriers was assessed by measuring the trans-endothelial resistance (TEER) over time. TEER measurement is a widely accepted quantitative technique for evaluating the integrity of cellular barriers in vitro [59,60] and serves as a key method for assessing the functional changes in barrier integrity in eye disease models, such as diabetic retinopathy [38], ocular hypertension [61], and wet age-related macular degeneration [62], among others. The TEER of the confluent cell barriers was measured daily for 7 days using an epithelial voltmeter EVOM2 (Fisher Scientific, NC9792051, Carlsbad, CA, USA). The EVOM2’s probe (Fisher Scientific, NC9679852, Carlsbad, CA, USA) was first calibrated in warm media to reach a baseline TEER value. The baseline number was subtracted from the measured TEER values in each test well. At the end of the 7 days, the cell viability across all the groups was assessed with a LIVE/DEAD™ Cell Imaging Kit (Thermo Fisher, R37601, Waltham, MA, USA) to confirm that cell density was maintained and that any changes in the TEER reflected the barrier function rather than cell death, as shown in Supplementary Figure S1.

### 4.9. TNF-α Treatment

Treatment with tumor necrosis factor alpha (TNF-α) was administered to the cell barriers in the Transwell inserts to measure the ability of the cell barriers to restore the values of resistance to the basal levels. TNF-α was selected because it is an inflammatory cytokine known to reduce cell barrier integrity [63,64]. The cultures of the MG, ECs, HMG, and HECs in the well plates were exposed to TNF-α (Thermo Fisher, 400-14-5UG, Waltham, MA, USA) and diluted in basal media at 1 ng/mL, 5 ng/mL, or 10 ng/mL for 48 h. The TNF-α concentrations were chosen using previous studies that demonstrated the concentrations caused the disruption of the barrier integrity in the Transwell cultures of endothelial cells [64,65]. A 5 ng/mL concentration of TNF-α was chosen for the recovery assays because it demonstrated visible phenotypic changes in cell viability.

### 4.10. Anti-VEGF (ARVA) Treatment 

An anti-VEGF treatment was also administered to the cell barriers formed in the Transwell inserts to measure the impact of VEGF-A inhibition on the cell barrier resistance. This study used a rat anti-VEGF-A (ARVA) molecule (Leinco Technologies, V142, Fenton, MO, USA) that operates in a similar fashion as bevacizumab, an anti-VEGF-A agent used to treat aberrant angiogenesis in humans [66]. ARVA, at a concentration of 1 μg/mL, was reconstituted in basal media and administered to MG, HMG, ECs, and HECs cultured in wells, as per the literature [43,44].

### 4.11. Measurement of TEER in TNF-α and ARVA Groups

The TEER of the confluent cell barriers was measured once a day for the first 3 days. On the third day, a solution of TNF-α at 5 ng/mL or ARVA at 1 μg/mL diluted in media was added to the apical (top) and bottom side of the Transwell of each cell barrier group, followed by TEER measurements at 1 h, 3 h, 6 h, and 24 h. After recording the 24 h time point, the treatment solution from each Transwell insert and was removed and the Transwell inserts were placed in DPBS for 2 min. Fresh media was replaced with each of the group’s respective media (basal or hyperglycemic) and the TEER was recorded until day 6 of the study. Barrier recovery was measured by the percent change in the TEER from the last treatment time point (24 h) to the measured TEER value on day 6.

### 4.12. Imaging and Software

An epifluorescence microscope (Leica DMi8, Chicago, IL, USA) with a cooled CCD camera (DFC7000 GT, Leica, Chicago, IL, USA) and LAS X Science microscope software Version 5.2.2 was used to capture the images in both the brightfield and in fluorescence via 10× or 20× objective. The fluorescence intensity was quantified using DAPI, GFP, TXR, and CY5 filters matching the corresponding fluorophore for the immunocytochemistry studies.

### 4.13. Statistical Analysis and Software

Two-way ANOVA was used to analyze the statistical significance among the groups at different time points (e.g., TEER assays). A two-way ANOVA repeated measures was used to determine the effect of the hyperglycemic medium on cell area over time on the treated groups (e.g., cell area change in HMG from day 1 to day 6). A one-way ANOVA was also used to assess the parametric data from single-time point studies (e.g., immunocytochemistry). A post hoc Tukey test was performed to identify the level of statistical significance among the groups. Each study included a minimum of 45 cells with at least 3 replicates per experimental condition for the cell morphology studies. Immunocytochemistry assays used at least 10 cells from 5 different regions (grid) of the well with 3 replicates per condition. The cell barrier assays included at least 3 replicates per condition with 3 readings per replicate from different regions of the Transwell. The statistical significance was denoted by symbols: * or †, where *p* < 0.05 = * or †; *p* < 0.01 = ** or ††; *p* < 0.001 = ***; *p* < 0.0001 = ****; n.s. = not statistically significant. All the statistical tests were performed using GraphPad Prism 10 software.

## 5. Conclusions

The effects of the AGEs from chronic hyperglycemia are well-known to cause cell apoptosis and dysfunction that are compounded in barrier tissue. This study illustrates significant cell and molecular relationships between the cell barriers of Müller glia and endothelial cells critical to the response and function of the inner blood retinal barrier in hyperglycemia. Our data illustrate the unexplored impacts of Müller glia communication with endothelial cells in barrier resistivity and highlight the significance of this glial vascular unit in the development of combinatory therapies for diabetic retinopathy.

## Data Availability

The original contributions presented in this study are included in the article/Supplementary Material; further inquiries can be directed to the corresponding author.

## Supplementary Materials

The following supporting information can be downloaded at https://www.mdpi.com/article/10.3390/ijms252212271/s1.
