# Supplementary figures and images for: Müller Glia Co-Regulate Barrier Permeability with Endothelial Cells in an Vitro Model of Hyperglycemia
Source: Int J Mol Sci. 2024 Nov 15;25(22):12271. doi: 10.3390/ijms252212271 (PMC11595118; doi:10.3390/ijms252212271)

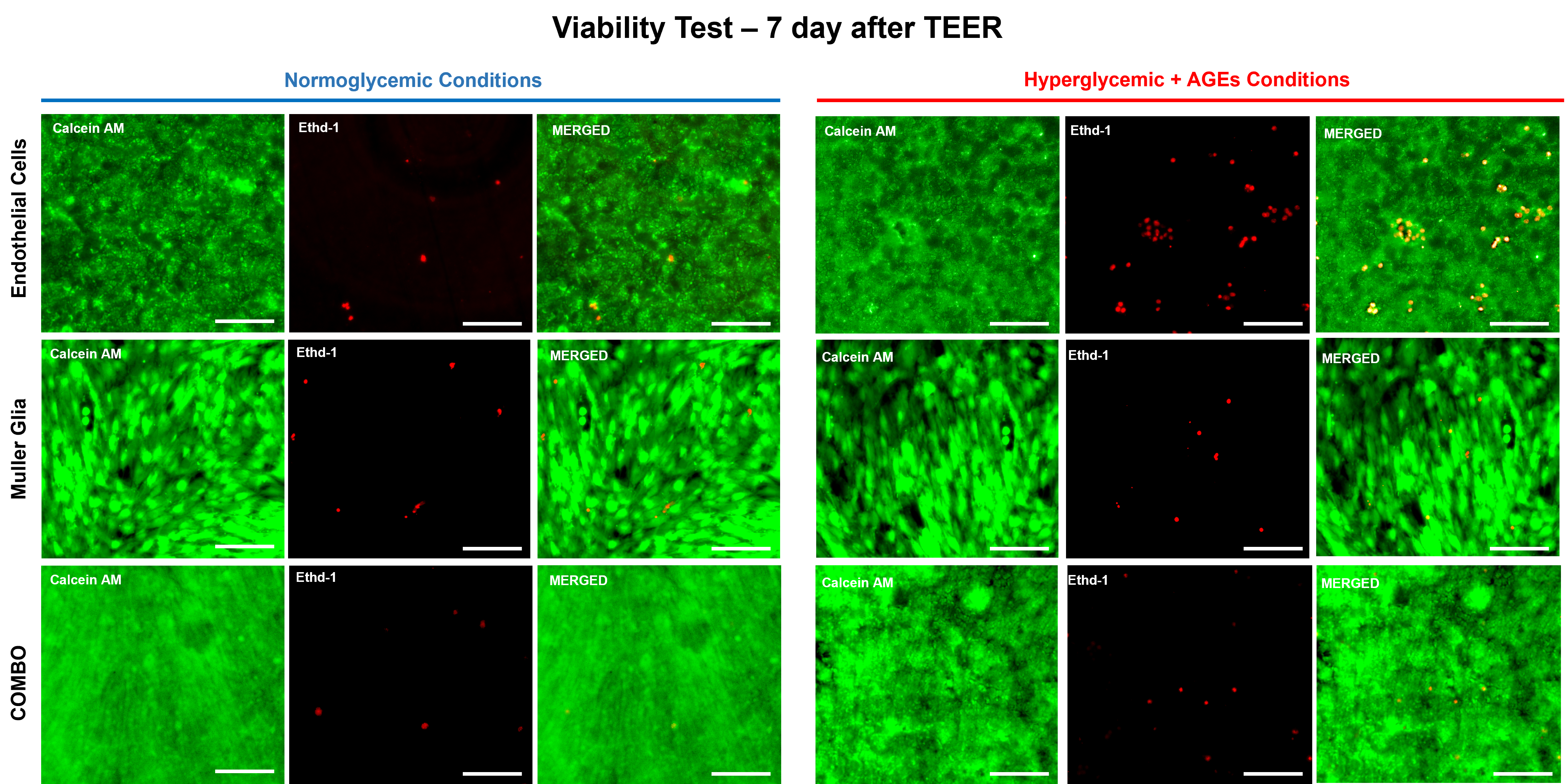

Supplement: Supplementary file 1 [file ijms-25-12271-s001.zip › ijms-3237644-supplementary.png]
